# Cellular Responses Induced by Zinc in Zebra Mussel Haemocytes. Loss of DNA Integrity as a Cellular Mechanism to Evaluate the Suitability of Nanocellulose-Based Materials in Nanoremediation

**DOI:** 10.3390/nano11092219

**Published:** 2021-08-28

**Authors:** Patrizia Guidi, Margherita Bernardeschi, Mara Palumbo, Vittoria Scarcelli, Massimo Genovese, Giuseppe Protano, Valentina Vitiello, Lorenzo Pontorno, Lisa Bonciani, Isabella Buttino, Gianluca Chiaretti, David Pellegrini, Andrea Fiorati, Laura Riva, Carlo Punta, Ilaria Corsi, Giada Frenzilli

**Affiliations:** 1 Department of Clinical and Experimental Medicine-Section of Applied Biology and Genetics and INSTM Local Unit, University of Pisa, 56126 Pisa, Italy; patrizia.guidi@unipi.it (P.G.); margherita.bernardeschi@for.unipi.it (M.B.); m.palumbo@studenti.unipi.it (M.P.); vittoria.scarcelli@unipi.it (V.S.); massimo.genovese@student.unisi.it (M.G.); 2 Department of Physical, Earth and Environmental Sciences and INSTM Local Unit, University of Siena, 53100 Siena, Italy; giuseppe.protano@unisi.it (G.P.); ilaria.corsi@unisi.it (I.C.); 3 Italian Institute for Environmental Protection and Research (ISPRA), Via del Cedro, 38, 57123 Livorno, Italy; valentina.vitiello@isprambiente.it (V.V.); isabella.buttino@isprambiente.it (I.B.); gianluca.chiaretti@isprambiente.it (G.C.); david.pellegrini@isprambiente.it (D.P.); 4 Biochemie Lab. S.r.l., Via di Limite 27G, 50013 Campi Bisenzio, Italy; l.pontorno@biochemielab.it (L.P.); l.bonciani@biochemielab.it (L.B.); 5 Department of Chemistry, Materials, and Chemical Engineering “G. Natta” and INSTM Local Unit, Politecnico di Milano, 20131 Milano, Italy; andrea.fiorati@polimi.it (A.F.); laura2.riva@polimi.it (L.R.)

**Keywords:** *Dreissena polymorpha*, freshwater nanoremediation, ecofriendly nanomaterials, polysaccharide-based materials, zinc bioaccumulation, acute toxicity, genotoxicity, DNA damage, micronucleus

## Abstract

Zinc environmental levels are increasing due to human activities, posing a threat to ecosystems and human health. Therefore, new tools able to remediate Zn contamination in freshwater are highly recommended. Specimens of *Dreissena polymorpha* (zebra mussel) were exposed for 48 h and 7 days to a wide range of ZnCl_2_ nominal concentrations (1–10–50–100 mg/L), including those environmentally relevant. Cellulose-based nanosponges (CNS) were also tested to assess their safety and suitability for Zn removal from freshwater. Zebra mussels were exposed to 50 mg/L ZnCl_2_ alone or incubated with 1.25 g/L of CNS (2 h) and then removed by filtration. The effect of Zn decontamination induced by CNS has been verified by the acute toxicity bioassay Microtox^®^. DNA primary damage was investigated by the Comet assay; micronuclei frequency and nuclear morphological alterations were assessed by Cytome assay in mussels’ haemocytes. The results confirmed the genotoxic effect of ZnCl_2_ in zebra mussel haemocytes at 48 h and 7-day exposure time. Zinc concentrations were measured in CNS, suggesting that cellulose-based nanosponges were able to remove Zn(II) by reducing its levels in exposure waters and soft tissues of *D. polymorpha* in agreement with the observed restoration of genetic damage exerted by zinc exposure alone.

## 1. Introduction

Among environmental contaminants, metals have gained attention because of their toxic and harmful effects on aquatic organisms and human health, also being able to induce genotoxicity through different mechanisms. Zinc (Zn) is an essential element for life, the background level of which in river waters varies from less than 10 to 200 µg/L [1]. However, increasing Zn concentrations into the environment due to human activities (e.g., mining and industrial activity, agriculture, and traffic) represent a serious concern for ecosystems and human health. In terms of cellular toxicity, Zn has been reported to affect DNA by generating reactive oxygen species (ROS) or by directly binding to DNA and/or to impair the repair enzymes in marine bivalve [2].

Nano-based technology (nanotechnology) has dramatically increased over the last years and has influenced many areas of modern life, including cosmetics, pharmaceuticals, and medical devices [3]. Nanotechnology also makes use of nanomaterials for removing pollutants from environmental matrices (nanoremediation) [4]. Being more effective and less expensive than conventional in situ remediation techniques, nanoremediation is proposed as an environmentally and economically sustainable new technology [5,6]. 

Nanomaterials employed in remediation processes, such as TiO_2_ nanoparticles (NPs), ZnO NPs, and carbon nanotubes, have been reported to exert a certain degree of toxicity to the biota [7,8,9,10,11,12]; moreover, environmental fate of nanoparticles, after their release into contaminated areas, may cause potential risks and potential ecotoxicity [13].

Recently, sustainable cellulose-based nanomaterials are becoming increasingly attractive for their potential use in environmental remediation [14,15,16,17,18,19]. In this background, ecotoxicology can provide suitable tools able to select eco-safe and sustainable nanomaterials for environmental remediation [20].

In our previous works, we described the ability of a cellulose-based nanosponge (CNS) to efficiently remove organic pollutants (e.g., dyes, drugs, and other organic compounds) [21,22,23] and inorganic pollutants (e.g., Cd(II), Cr(III), Cu(II), Hg(II), Ni(II), Co(II), and Zn(II) ions) [17,23] from contaminated water. In addition, we observed reduced genotoxicity caused by bivalent metals such as Zn in marine mussels (*Mytilus galloprovincialis*) [18] and Cd in zebra mussels (*Dreissena polymorpha*) [19]. Based on that, in the present study we aimed to test the ability of CNS to reduce zinc genotoxicity in zebra mussel haemocytes. The bivalve *D. polymorpha* was the model chosen to test the efficacy of zinc genotoxicity prevention as well as ecosafety of CNS based on its ability to remove heavy metals from freshwater. Zn(II) concentration in exposure waters and in soft tissues of zebra mussels were investigated before and after CNS treatment at time 0 and 48 h of exposure. Zn concentrations in CNS themselves before and after contact with Zn (50 mg/L) were also measured, as well as acute toxicity. Genotoxic potential was evaluated at the molecular level by the Comet assay and at the chromosomal level by Cytome assay; acute toxicity was assessed by bioassay Microtox^®^ using *Aliivibrio fischeri*.

## 2. Materials and Methods

### 2.1. Reagents and Equipments

ZnCl_2_ (CAS 7646-85-7), low and normal melting point agarose, Neutral Red, phosphate buffer saline were supplied by Sigma Aldrich (Milano, Italy), Giemsa by Carlo Erba (Milano, Italy). Cellulose from cotton linters was kindly provided by Bartoli SPA (Capannori, Lucca, Italy). Deionized water (DI) was prepared with a Millipore Elix^®^ Deionizer equipped with a Progard^®^ S2 ion exchange resins. The other equipment employed are: Branson Sonifier 250 (Branson Ultrasonic SA, Carouge, Switzerland), 6.5 mm probe tip and a SP Scientific BenchTop Pro Lyophilizer (Perugia, Italy) and a Transmission Electron Microscope (TEM; Philips CM 200, Koninklijke Philips N.V., Amsterdam, The Netherlands). Zeiss EVO 50 EP was provided by Carl Zeiss Microscopy Gmb (Jena, Germany). Milestone Ethos 900 microwave lab station (Sorisole, Italy). Lyophilised bacteria, all Microtox^®^ reagents, Microtox^®^M500 luminometer and Microtox OMNI™ v. 1.16 software were obtained from ECOTOX LDS (Cornaredo, Milan, Italy). Coupled Plasma-Optical Emission Spectrometry (ICP-OES) was performed with Perkin Elmer Optica 8300 (Perkin Elmer, Waltham, MA, USA). Zn determination in soft tissues and CNS was carried out by inductively coupled plasma-mass spectrometry (ICP-MS) using the Perkin Elmer NexION 350 spectrometer (Waltham, MA, USA).

### 2.2. Preparation and Characterization of the Cellulose-Based Nanosponge (CNS)

#### 2.2.1. Synthesis of TEMPO-Oxidized Cellulose

Cellulose was oxidized as previously reported [24,25,26]. Briefly, cellulose (100 g) was dispersed in DI water (5.7 L) and 2,2,6,6-tetramethylpiperidine-1-oxyl (free radical) (TEMPO, 2.15 g, 13.8 mmol) and KBr (15.42 g, 129 mmol) were dissolved in the suspension. Then, an aqueous solution of NaClO (12.5% *w/w*, 437 mL) was dropped to the slurry under vigorous stirring. During the reaction, the pH was kept in the range of 10.5–11 by addition of NaOH_aq_ (4 M). The reactive mixing was stirred overnight at room temperature. Then, HCl_aq_ (12 M, 5 mL) was added in order to coagulate the oxidized cellulose, which finally was collected by filtration and intensively washed with DI (6 × 550 mL).

The amount of carboxyl groups was evaluated by a colorimetric titration, exploiting phenolphthalein as colorimetric indicator. An oxidation degree of 1.5 mmol_COOH_/g_TOCNF_ was measured.

#### 2.2.2. Synthesis of CNS

CNS were prepared as previously reported [17,21]. In brief, 3.5 g of oxidized cellulose were suspended in DI (2.5% *w/v*), and a stoichiometric amount of NaOH was added. The dispersion was ultrasonicated, in order to promote the separation of the TEMPO oxidized cellulose nanofibers (TOCNF), until the obtainment of a homogeneous and clear dispersion. The TOCNF dispersion was acidified with HCl_aq_ (2 M), recovered by filtration and washed with DI until the percolated water reached pH 6–7. Then, 10 mL of an aqueous solution of bPEI (2.5 KDa, 0.35 g/mL) and 10 mL of aqueous citric acid (CA) solution (0.0896 g/mL) were mixed with the TOCNF dispersion, under continuous stirring, obtaining a homogeneous hydrogel. This hydrogel was transferred in 24-plate multiwells, frozen at −35 °C, and freeze-dried for 48 h. The achieved xerogels were removed from the mold and thermally treated at 102 °C for 16 h. The obtained CNS was ground in order to provide a fine powder and washed with DI water (6 × 40 mL) and ethanol (1 × 40 mL). 

#### 2.2.3. Characterization of TOCNF and CNS

The morphological characterization of TOCNF was obtained by a transmission electron microscope operating at 200 kV and equipped with a field emission gun filament. Scanning electron microscopy (SEM) and SEM-EDS observations were carried out using a Zeiss EVO 50 EP scanning electron microscope (Zeiss International, Oberkochen, Germany) connected with a Bruker Quantax 200 6/30 detector (Bruker, Billerica, MA, USA) for Energy-dispersive X-ray spectroscopy (EDS) analysis (SEM Cambridge Stereoscan 360). The acceleration voltage was set to 20 kV with an electron beam current intensity of 100 pA, operating at a working distance of 8.5 mm. The specimens were used without any treatment.

### 2.3. Sampling and Maintenance Condition

*Dreissena polymorpha* specimens (medium valves length 2.01 ± 0.5 cm) were sampled from an artificial lake (Bilancino Lake, Tuscany, Italy), characterized by a Zn(II) concentration of 0.05 ± 0.001 mg/L. Mussels, in 3 independent samplings, were carried to the laboratory in lake original water and were placed 5 days before the dose-exposure experiment and 2 days before the co-exposition experiments in aquaria (10 L) containing aerated artificial freshwater (AFW) made of de-chlorinated tap (50%) and distilled water (50%) for the acclimatization period. A natural photoperiod was maintained and water temperature was 18 ± 1 °C, pH was 7.59 ± 0.36 for exposure experiments to zinc and 8.07 ± 0.39 for combined experiments where artificial freshwater contaminated by Zn(II) was combined with CNS. Independent experiments were carried out.

### 2.4. In Vivo Exposure

At the end of the acclimatization period, specimens of *D. polymorpha* were located on glass sheets placed in glass aerated tanks. The experimental design planned at least 25 specimens for each group. Two different exposure times were included: 48 h for the Comet assay and 7 days for the Cytome assay. Animals fasted until being sacrificed, and only mussels re-attached by their byssus on glass sheets under the water were selected for the analysis [27]. Mussels were exposed to 1, 10, 50, and 100 mg/L Zn(II) nominal doses for 48 h and 7 days of select sub-lethal concentration for the CNS efficacy combined study.

A stock solution was set up by dissolving ZnCl_2_ in distilled water. Final concentrations were prepared in AFW and chosen on the basis of these preliminary results and data from the literature [28], in order to work with environmentally realistic exposure levels. The threshold limit established by the Italian Minister for groundwater is actually 3 mg/L, as well as the one for drinking water [29] and spring water [30], so lower and higher doses were selected. Moreover, combined experiment selected dose is representative of polluted water river, as in the Brazilian Toledo River, where the most impacted site was characterized by peaks of 50 mg/L Zn water concentration [31]. The Zn(II) doses found to be genotoxic but not cytotoxic were 10 and 50 mg/L. Thus, also taking into account environmentally relevant concentrations [31,32] 50 mg/L nominal dose was selected to test CNS.

The experiments aimed to assess CNS eco-safety and zinc adsorption efficacy in freshwaters were thus conducted (combined experiments). For this purpose, freshwater bivalves were exposed (48 h) to the following groups in AFW: 50 mg/L ZnCl_2_ (Zn(II)), 50 mg/L ZnCl_2_ after treatment with CNS (Zn-t CNS), AFW after treatment with CNS alone (CNS). Controls were represented by specimens in solely artificial freshwater. Based on our previous study [18], the ratio of CNS able to adsorb Zn(II) from fresh waters was set up at 1.25 g of CNS in 1 L of artificial freshwater. A strong magnetic stirring (2 h at room temperature) was exerted on the CNS samples in AFW to reproduce the protocol usually employed for polluted water. 0.45 µm filters were used to remove CNS powder from the conditioned AFW and the bivalve specimens were exposed to the resulting water. The potential genotoxic effects of Zn(II) and CNS-treated AFW, alone and in combination, were assessed in *D. polymorpha* haemocytes. Cells were kindly aspirated from the posterior adductor muscle sinus and immediately employed for the Comet and Cytome assays [33]. Twenty-five specimens from each experimental group were used for biochemical analysis.

### 2.5. Zinc Concentration in Water

Chemical analyses were performed on water samples collected from each aquarium immediately after the treatment and at the end of the exposure time (48 h) and then stored at 4 °C. Zinc concentrations were measured through an Inductively Coupled Plasma-Optical Emission Spectrometry (ICP-OES) using a Perkin Elmer Optica 8300 (PerkinElmer, Inc., Waltham, MA, USA), equipped with a CrossFlow nebulizer and a Scott Spray Chamber, followed by a standard quartz torch. The instrument calibration was performed by dilution of a zinc analytical standard (FLUKA), with MilliQ^®^ water (Merk Life Science, Milano, Italy) obtaining 5, 10, and 50 µg/L solutions; to each analyzed samples, Y (2 mg/L) was added as internal standard.

### 2.6. Zn Concentrations in CNS and Zebra Mussel

Zinc concentrations were measured in CNS (1.25 g/L) after 2 h of incubation in AFW and in AFW with 50 mg/L Zn(II), and in whole soft tissues of *D. polymorpha* at the end of the exposure time (48 h) in all the tanks: control (AFW), AFW treated with CNS only (CNS), 50 mg/L Zn(II) in AFW treated with CNS (Zn-t CNS), 50 mg/L ZnCl_2_ in AFW (Zn(II)).

CNS were collected from the filters used to remove them from exposure waters and dried at +30 °C in a ventilated oven. Ten individuals of zebra mussel from each experimental group were freeze-dried after removing the valves using a Labogene, Scanvac (Labogene, Nyköping, Sweeden) cool safe (0.5 torr pressure, T= −50 °C).

CNS and zebra mussels’ whole soft tissues were solubilized by means of microwave-assisted acid digestion by adding 3 mL HNO_3_ and 0.5 mL H_2_O_2_ (ultrapure reagents) to about 250 mg of sample. Solubilization was carried out in Teflon bombs in a Milestone Ethos 900 microwave lab station (Milestone, Sorisole, Italy).

Determination of Zn concentrations in CNS and zebra mussel was performed by inductively coupled plasma-mass spectrometry (ICP-MS). To assess analytical accuracy, the following standard reference materials with certified Zn concentrations were analyzed: GBW 07604 (Poplar Leaves) of Institute of Geophysical and Geochemical Exploration (Langfang, China) and SRM 2977 (Mussel Tissue) of National Institute of Standards and Technology (Gaithersburg, MD, USA). Zn recoveries were 99.1 and 100.3% for GBW 07604, 99.3, and 99.6% for SRM 2977. The precision of Zn measurements was defined through the percentage relative standard deviation (% RSD) of five replicate analyses of Zn(II) in each CNS and zebra mussel sample. The values of % RSD were below 0.7%.

### 2.7. Viability Assessment

The Neutral Red Retention Time (NRRT) assay was used as a measure of cytotoxicity due to its recommended use for regulatory cytotoxicity evaluation of chemicals by OECD and NIH, USA [34] and widely used to assess cell viability. It is used to distinguish cells actively retaining the neutral red dye inside their lysosomal membranes from cells unable to do it. Neutral Red Retention Time assay was performed according to Guidi and collaborators [33]. The Comet assay was applied only on haemocytes that displayed a cell toxicity <10% in order to exclude potential false positive data.

### 2.8. Comet Assay

The Comet assay was carried out on haemocytes from 10 zebra mussels taken from each experimental group at the end of the exposure time, according to Guidi et al. [35]. During haemolymph collection, individual cell suspensions were stored at +4 °C in the dark and then samples were centrifuged for 10 min at 125× *g*. Cell pellet was embedded in 75 µL of freshly made 0.5% LMA and spread on microscopy glass slides, pre-coated with a layer of 1% NMA. The second layer agarose polymerization was allowed for 5 min on metal trays at +4 °C, and then an additional layer of 85 µL of 0.5% LMA was added. Following agarose solidification at +4 °C, slides were immersed in freshly made lysing solution (10 mM Tris, 2.5 M NaCl, 1% Triton X 100, 0.1 M EDTA and 10% DMSO, pH 10) for at least 1 h. To allow DNA unwinding in alkaline conditions, a horizontal gel electrophoresis chamber was used to incubate slides for 10 min at +4°C with fresh electrophoresis buffer (0.075 M NaOH, 1 mM EDTA, pH ≥ 13). Electrophoresis was performed at 25 V, 300 mA, for 5 min at +4 °C. To neutralize the pH, and allow DNA staining, at the end of the electrophoresis run, slides were washed three times (5 min each) with a neutralization solution (Tris-HCl, pH 7.5). Slides were stained with ethidium bromide and scored under a fluorescence microscope (400×). An image analyzer (Kinetic imaging, Ltd., Liverpool, United Kingdom, Komet, Version 5) was used and the parameter chosen to quantify the amount of DNA damage was the percentage of DNA migrated into the comet tail (% tail DNA) [36]. At least 50 randomly chosen nuclei per slide and 2 slides per sample were scored, for a total of 100 nuclei per organisms and the mean calculated.

### 2.9. Cytome Assay

Chromosomal damage was evaluated by assessing the frequency of micronucleated haemocytes and nuclear abnormalities. The cytome assay was performed according to Guidi et al. [33]. For chromosomal damage evaluation, 500 well-preserved cytoplasm haemocytes per specimen were analyzed at light microscope, as suggested by Fenech (2007) [37]. These nuclear morphology alterations (NA) were included: nuclear blebs (BL), nuclear buds (NBUD), notched nucleus (NT), circular nucleus (CIR), lobed nucleus (LB), nucleoplasmic bridges (NPB), anisochromatic nuclei (AN), and apoptotic cells (APO). NPB are the expression of the chromosomal mutation “dicentric chromosome” coming from the joint of two broken chromosomes. For each experimental group 10 animals, 2 slides per animal, 500 cells per slide were observed. 

### 2.10. Acute Toxicity Test with Aliivibrio Fischeri

Water samples were taken from the controls (AFW), CNS and Zn-t CNS treatments immediately (t0) and at the end of the exposure time (48) h, and stored at −20 °C until the analysis. Previously, an acute test with *Aliivibrio fischeri* exposed to the nominal dose of 50 mg/L Zn(II) solution was performed to evaluate EC50. After reactivation, bacteria were added in each samples dilutions and incubated at 15 °C using Microtox M500 luminometer. Bacteria light emission was recorded for each dilution and replicated at the beginning of incubation and after 5, 15, and 30 min of exposure.

Data were analyzed using the MicrotoxOmni™ software (version, 4.2, Modern Water, London, UK), which calculated Maximum Effect percentage for each exposure time and effect concentrations EC20 and EC50 when low luminescence values are recorded by the luminometer. The acute toxicity test was determined following the Basic 90% Protocol ISO 11348 [38], with seven sample dilutions and three replicate of controls. Toxicity was calculated by using maximum effect percentages or EC20 and EC50 values, in accord with ICRAM-APAT 2007 [39].

### 2.11. Statistical Analysis

Data from at least 5 animals were analyzed by a statistical software Statgraphics Centurion XV (version 15.1.02, by StatPoint, Inc., Warrenton, VA, USA) from each experimental point. Being an in vivo study, each animal was considered as a statistical unit, then mean ± SD was considered per experimental group. Results are represented as mean ± SD after multifactor analysis of variance (MANOVA). In order to investigate differences among the experimental groups, the multiple range test (MRT) was applied. For all the analyses, a statistical significance level of *p* < 0.05 was set up [40].

## 3. Results and Discussion

The main purpose of this study was to investigate the safeness and efficacy of CNS obtained by renewable sources in preventing zinc-induced genotoxicity in zebra mussel haemocytes.

### 3.1. Dose-Effect Experiments

**Figure 1:**
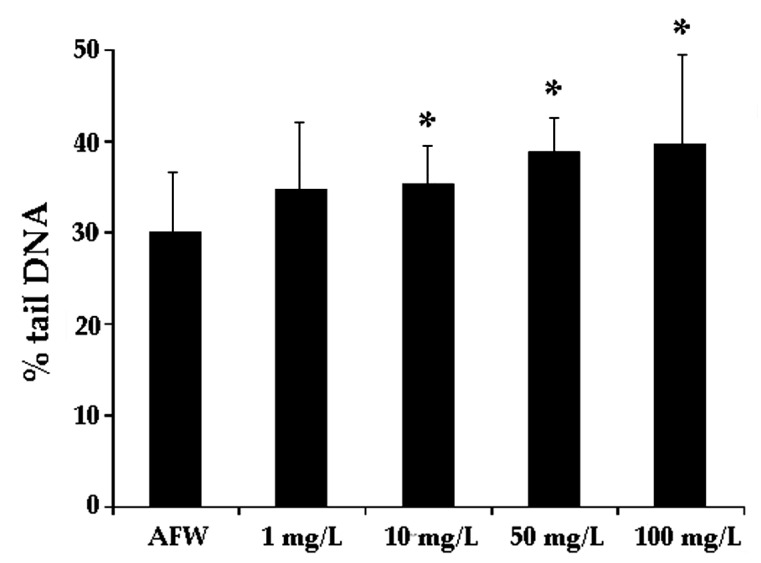
DNA primary damage (% tail DNA) in zebra mussels’ haemocytes after 48 h of exposure to the following experimental groups: AFW (control); 1–10–50–100 mg/L Zn(II)nominal doses in AFW. Data are shown as mean ± SD. (*) indicates significant difference respect to the control group (AFW) (*p* < 0.05).

In order to select a genotoxic and sub-cytotoxic dose evaluated by the Comet assay to be used for the co-exposure investigations with CNS, zebra mussels were exposed to different concentrations of Zn(II). A loss of DNA integrity was detectable (*p* < 0.05) at the nominal doses of 1, 10, 50, and 100 mg/L Zn(II) after 48 h exposure (Figure 1). Cytotoxicity was observed at the nominal dose of 100 mg/L (data not shown). However, we verified that starting from the 50 mg/L Zn(II) solution in AFW, a white precipitate occurred after a few minutes. We assumed this behavior could be ascribed to the presence of carbonates in AFW, containing 50% of de-chlorinated tap, which would lead to the formation of low soluble zinc carbonates. As a matter of fact, Zn(II) concentration in this case suddenly decreased in a range between 20–30 mg/L for the nominal dose of 50 mg/L and in a range between 35.5–47.6 mg/L for the nominal dose of 100 mg/L. Nevertheless, even in this range, genotoxic action of Zn(II) was observed, as reported in Figure 1. Although the background level of DNA damage in control zebra mussels obtained in the present study does not overlap with recommended values suggested by Tice and co-authors [41], data from literature reported high background levels obtained by the Comet assay in *D. polymorpha* and in other aquatic organisms [19,42]. Baseline levels of DNA damage in zebra mussel haemocytes were also found to correlate with animal maintenance temperature [43]. We also cannot ignore that results obtained from AFW experimental group might have been modulated by other factors such as water quality of sample site and/or origin population genetic background. It is important to underline that the results obtained from the control tank were able to discriminate the exposure effects indicating a DNA integrity degree acceptable for a control sample.

The sensitivity of zebra mussels to genotoxic agents has been demonstrated through the induction of micronuclei and DNA strand-breaks [44,45]. Similarly, field studies revealed an increase of DNA damage in haemocytes of zebra mussels inhabiting polluted sites, which correlated with the level of contaminants in water [27]. In the present study in vivo zinc exposure induced a statistically significant increase of DNA strand breaks compared to controls, confirming data from the literature evaluated in different sentinel species [46,47,48], being Zn induced DNA damage also known to occur in marine mussels’ cells [18,49,50].

In terms of chromosomal damage and nuclear abnormalities, an induction of micronucleated cells (*p* < 0.05) and nucleoplasmic bridges (*p* < 0.05) were assessed after the exposure to highest dose of Zn(II). Apoptotic nuclei were observed in the absence of necrotic events. Treatment groups were not statistically different from the control in terms of apoptosis (Table 1).

Zinc is also known to induce chromosomal damage in aquatic species [51]. In gill cells, Majone et al. [52] found that mussels exposed under controlled laboratory conditions to ZnCl_2_ showed a clear increase in MN frequencies after treatment, suggesting that this metal even at low concentration (0.17 mg/L) and in short term exposure (48 h) exerts a clear clastogenic activity. The presence of nucleoplasmic bridges coming from two clastogenic events seems to confirm this mechanism of action possessed by zinc. As reported in the literature, nuclear abnormalities have been used as a marker of genotoxicity in marine mussels’ cells [53], even related with heavy metal exposure [54]. In literature, the majority of data regarding apoptotic cell frequency in zebra mussel was obtained through the diffusion (or halo) assay. Values ranged between 2 and 4 apoptotic nuclei per 100 cells scored, which means they represent about the 2% of the cell population analyzed [55,56]. These values are similar to those reported here, even if they originated from Cytome assay slides analysis, since the relationship between the apoptotic nuclei scored and the total amount of cells scored is quite the same (7–14/1000 cells).

### 3.2. Combined Experiments

#### 3.2.1. Synthesis and Characterization of Cellulose Nanosponges

**Figure 2:**
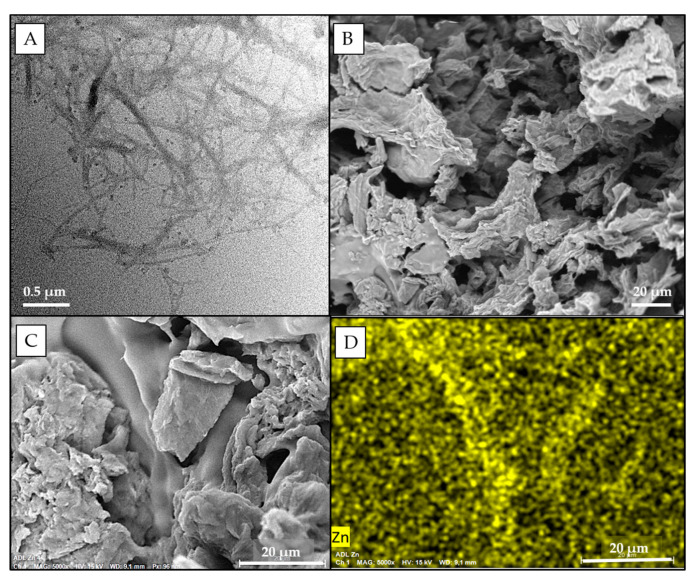
(**A**) TEM image of TOCNF; (**B**) SEM image of CNS powder before zinc decontamination activity; (**C**) SEM image of CNS powder after Zn adsorption; (**D**) SEM-EDS image of CNS after Zn adsorption clearly showing the presence of Zn on the nanosponge (yellow dots).

**Figure 3:**
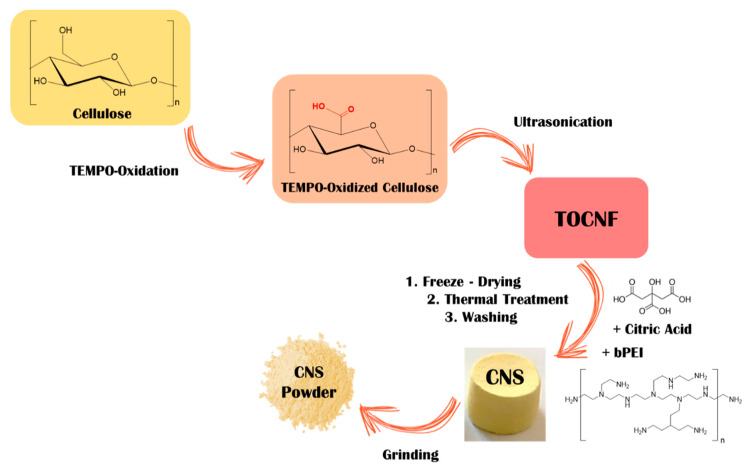
Synthesis of cellulose-based nanosponges (CNS).

TEM analysis of TOCNF confirmed the expected morphology of the fibers [25], characterized by a micrometric length and nanometric width (Figure 2A). These nanofibers were used as building blocks for the production of cellulose-based nanosponges (CNS), which were synthesized according to Figure 3, following a multistep procedure previously optimized [17]. The formulation required a 1:1 weight ratio between TOCNF and bPEI and the addition of 18% of CA with respect to primary amino groups of bPEI. CA is needed to increase the bPEI crosslink within the final network.

A detailed and fully comprehensive characterization of CNS was reported in previous works. In particular, the FT-IR analysis evidenced the presence of the −C=O stretching vibrational mode of the amide functional groups (peak at 1664 cm^−1^), while the ^13^C CP-MAS solid-state NMR and the elemental analysis also confirmed the role of CA in better fixing bPEI in the network [22,57,58].

An SEM image of CNS reported in Figure 2B confirms the expected high micro-porosity of the system, due to the pristine formation water ice crystals, with a two-dimensional sheet-like morphology and a pore size in the range of 10–100 micrometers. Moreover, the microcomputed tomography (μ-CT) analysis reported in Fiorati et al. [22] indicated that CNS possesses a trabecular inner structure, which has an average trabecular thickness of about 30–40 μm, a trabecular separation of about 70–75 μm, and a porosity of 70–75%.

Besides the evident micro-porosity of the network, a recent analysis of water nano-confinement geometries in CNS, conducted by small angle neutron scattering (SANS) technique, provided the first experimental evidence of nano-dimensioned porosity in the sorbent material, allowing a measurement of short-range correlation length in the range between 25 and 35 Å [57]. Moreover, an in-depth FTIR-ATR investigation of CNS hydrated with H_2_O and D_2_O allowed supporting the findings of a nano-confinement of water by detecting a supercooled behavior on the entrapped water molecules [58].

#### 3.2.2. Zn(II) Adsorption Efficiency of CNS

Results of the ICP-OES analysis on all the experimental points are reported in Table 2. In control (AFW) and AFW treated with CNS (CNS), the Zn(II) concentration was found to be insignificant. Different from what is observed above, Zn(II)-contaminated AFW (50 mg/L) treated with 1.25 g/L CNS (Zn-t CNS) showed a significant decrease in Zn concentration (up to 94%) in comparison with Zn(II) contaminated AFW (Zn-t AFW). It is to note that in the Zn-t CNS experimental group, the level of Zn(II) in zinc-contaminated water decreased to the values authorized by Italian law (3 mg/L) for drinking water.

Looking at Zn levels in nanosponges recovered after 2 h in AFW alone (AFW) and AFW contaminated by Zn (Zn-t CNS), the adsorption efficacy of CNS towards Zn was confirmed. In fact, Zn concentration of 365.23 ± 177 µg/g was measured in CNS from Zn-contaminated AFW, whereas Zn(II) level of 16.04 ± 0.11 was found in CNS alone in AFW. Moreover, SEM-EDS images of CNS powder from Zn-t CNS clearly proved the immobilization of Zn ions on the CNS (Figure 2D).

Interestingly, Zn concentration in the Zn(II) experimental group at T_0_ was found to be much lower than expected (~28 mg/L against the expected 50 mg/L). Moreover, after 48 h of exposure Zn(II), concentration in Zn-contaminated AFW further decreased, in respect to the sample analyzed soon after preparation (T_0_), suggesting a possible role of zebra mussels in filtering and accumulating the Zn ions.

For this reason, the amounts of Zn(II) in the whole soft tissue of zebra mussels were determined in all exposure groups. Indeed, level of Zn(II) in mussels exposed to Zn-t CNS (171.66 ± 0.44 µg/g d.w.) was significantly lower, compared to that in individuals exposed to only Zn (II) (505.5 ± 3.28 µg/g d.w.; Table 3).

**Figure 4:**
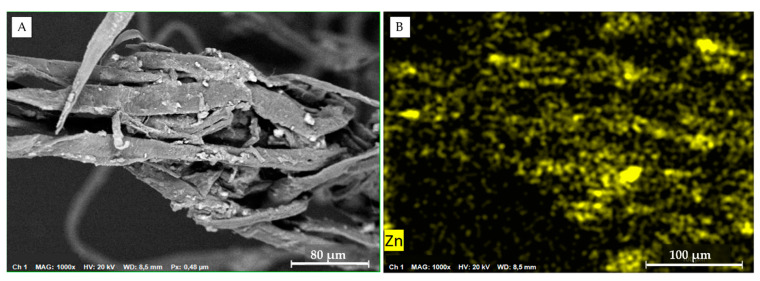
SEM images in *D. polymorpha’* feces: (**A**) SEM image with evidence of aggregates on the fibers; (**B**) SEM-EDS image of faces clearly showing the presence of Zn (yellow dots).

Zn(II) level in zebra mussels exposed to CNS alone (CNS) was similar to the control (AFW): 126.05 (±0.44) vs. 121.36 (±0.54) µg/g d.w. As further proof, Zn(II) body burden in zebra mussels collected from the Bilancino Lake before the acclimatation was 104.91 (±0.36) µg/g d.w. If, from one side, this result confirms again the effective role of CNS in removing Zn(II) from the AFW medium, the Zn(II) amounts found in mussels exposed to Zn (Zn(II)) did not justify the significant decrease in metal water concentration at T_48_. For this reason, we assumed that upon uptake by zebra mussel, Zn(II) is partially released in feces, as confirmed by SEM images thus explaining the low levels found in exposure waters (Figure 4).

Although the exposure period was only 48 h, it might be suggested that *D. polymorpha* is capable of regulating the body concentration of essential metals such as Zn [59]. More than half of the accumulated Zn was reported to be found in feces after 24 h [60] in *D. polymorpha*. Moreover, regulation is considered as an active process in the decapod *Palaemon elegans,* where an increased rate of Zn uptake was matched by an increase of Zn excretion [61], indicating the important role of bio-deposition, besides bioaccumulation. 

#### 3.2.3. Genotoxicity

**Figure 5:**
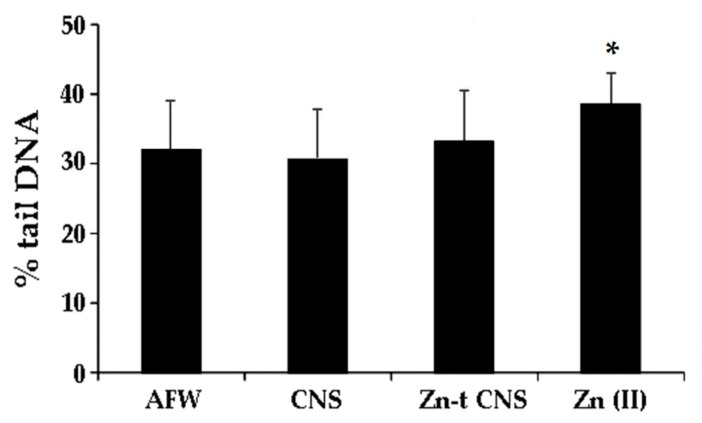
DNA primary damage (% tail DNA) in zebra mussels’ haemocytes after 48 h of exposure to the following experimental groups: AFW (control); Zn(II) (ZnCl_2_ 50 mg/L in AFW); Zn-t CNS (Zn(II) 50 mg/L) contaminated AFW treated with CNS), CNS (AFW treated with CNS only). Data are reported as mean ± SD. * indicates statistically (*p* < 0.05) significant differences respect to the control.

For the second set of experiments, the water samples were pretreated with CNS alone (CNS) or in combination with Zn(II) (Zn-t CNS) and the nominal dose of 50 mg/L (Zn50) was selected as genotoxic but not cytotoxic. Specimens exposed to AFW treated with CNS did not affect haemocytes’ DNA integrity. On the contrary, Zn50 induced a statistically significant increase in DNA primary damage, compared to the controls (*p* < 0.05). ZnCl_2_ in synthetic water previously flown through polysaccharide-based adsorbent CNS did not show any loss of DNA integrity in comparison with the control group, thus suggesting that polysaccharide-based nanosponges are able to soften ZnCl_2_ induced-DNA primary damage in zebra mussel haemocytes (Figure 5).

A relatively weak positive correlation was found between DNA primary damage and water Zn(II) concentration (*p* = 0.02; c.c. = −0.30; r^2^ = 9.3) while there is no statistically significant relationship between the frequency of micronucleated cells and Zn(II) concentration in exposure waters. There is a lack of investigation regarding correlations between DNA damage in aquatic specimens and water zinc concentration. In a polluted Brazilian river, Matos and co-workers (2017) [32] found higher genotoxic effects in fish sampled at the Zn most contaminated sites, but Zn levels were not directly correlated to toxicogenetic damage. On the contrary, Sargsyan and colleagues [62] found a positive correlation between DNA damage in *D. armeniaca* and content in the soil of Zn. Even if we are aware that researchers investigated a terrestrial animal, and the metal concentrations were detected in the soil, these data seem to support our observation. In terms of micronucleated cells frequency no statistically significant results were obtained after seven-day exposure (data not shown). Specimens exposed to AFW treated with CNS did not show any increase in micronucleated haemocytes. Similarly, no induction of nucleoplasmic bridges was found after the exposure to CNS in AFW only. With the present experimental approach, we were able to appreciate the suitability of CNS as Zn(II) removal in freshwater, which, besides the absence of adverse genotoxic effects, is able to prevent zinc-induced DNA integrity loss in *D. polymorpha* hemocytes, in agreement with previous findings on the marine bivalve *M. galloprovincialis* [18]. CNS themselves did not induce any DNA integrity loss, also at the chromosomal level. In fact, no statistically significant increase of in cells or nucleoplasmatic bridges were detected in specimens exposed to 1.25 g/L of CNS in artificial freshwater, compared to the control (data not shown). We remind that, despite an absence of genotoxic effects revealed by the nanomaterial (CNS) used in the present in vivo experimental conditions, the inhalation of nano-scaled cellulose may have adverse effects on human health, especially related to pulmonary exposure and because of its potential bio-persistence when inhaled [63]. However, there is a scarcity of chronic, low dose, and repeated exposure studies, making occupational exposure risk assessment of the various life stages of nanocellulose-containing products difficult [63].

#### 3.2.4. Acute Toxicity Test with *Aliivibrio fischeri*

In addition to the chemical and sub-lethal investigations conducted in order to determine the zinc concentrations and assess the safety and efficacy of CNS in preventing zinc induced genotoxicity, a bioassay with *Aliivibrio fischeri* was conducted. For this purpose, the water used for the co-exposure was tested to check for any qualitative toxicity during 48 h of incubation. Bacteria like *A. fischeri* is a well-known species that emits bright bioluminescence. The bioluminescence intensity is directly proportional to the metabolic activity of bacteria. Any inhibition of enzymatic activity can cause a decrease in bioluminescence. This property represents a simple way to measure the toxicity of different compounds on this biological system, which constitutes an important component of aquatic microbial community [64]. In Table 4, the results of the ecotoxicity tests with Microtox^®^ system carried out on water samples collected during co-exposure experiments are reported. 

Both AFW and CNS only in AFW did not induce any toxic effect on bacteria at both T_0_ and 48 h. Our test conducted with Zn(II) at the 50 mg/L nominal dose showed EC50 values of 85.45% (67.39–108.4, 95% Confident Interval C.I.), 8.665% (6.819–11.01, 95% C.I.), and 1.937% (0.8467–4.432, 95% C.I.), after 5, 15, and 30 min of incubation, respectively. These results confirm the high sensitivity of Microtox^®^ bioassay to heavy metals [65,66].

The effect of decontamination induced by CNS in zinc-contaminated water has been verified with bioassay with *A. fischeri*; both samples (T_0_ and 48 h) collected from the Zn-t CNS treatment had no effect on *bacteria* bioluminescence, with EC20 values up to 90% for each exposure time. 

*Aliivibrio fischeri* is considered to play an important ecological role, as it is at the base of most food webs and provides essential ecological and biochemical services, making it a good starting point for any ecotoxicity test [67]. Microtox^®^ is a standard government agency involved in ecotoxicological bioassay in Canada, The Netherlands, France, Germany, Spain, and Sweden. In the United States, it is involved in the Standard Methods for the Examination of Water and Wastewater [68,69] and in Italy, it is one of the bioassays suggested by the Italian Law on classification of dredged marine sediments [70]. Moreover, the Microtox^®^ bioassay has previously been shown to provide a good correlation with other species for a large number of chemical toxicants [71] and EC50 values recorded with this test have been correlated to acute toxicity parameters of vertebrates [69]. Furthermore, it is a simple, fast, robust, and cost-effective assay [68]. According to those characteristics, the bioluminescence test with *A. fischeri* is often chosen as the first test in ecotoxicological evaluations [72] and several studies have involved this test in toxicity evaluation of nanoparticles [67,73,74,75,76,77,78] and nanostructured materials [62,79,80].

## 4. Conclusions

Our results showed that Zn(II) induced DNA integrity loss and chromosomal mutation in zebra mussel hemocytes. CNS was found, for the first time, to be an effective eco-safe material for Zn genotoxicity prevention in light of their possible use in nanoremediation of freshwater environments. Indeed, in the present study, specimens exposed to ZnCl_2_ in synthetic freshwater previously flown through polysaccharide-based adsorbent nanosponges did not show any increase in DNA damage respect to the control, thus suggesting the efficacy of polysaccharide-based nanosponges in mitigating ZnCl_2_ induced-DNA primary damage in zebra mussel haemocytes. A significant decrease in Zn(II) water concentration was found at 48 h exposure. Such a concentration decrease was not justified by its bioaccumulation in the organisms. The detection of Zn(II) in zebra mussels’ feces highlighted the importance of bio-deposition in the active process of zinc regulation in *D. polymorpha*. 

The Microtox^®^ assay proved to be a valid and rapid tool for the eco-toxicological evaluation associated with remediation treatments with CNS. Moreover, the contemporary use of the acute toxicity test and sub-lethal endpoints enabled a better understanding of the safety and efficacy of nanomaterials obtained by renewable and sustainable sources.

## Figures and Tables

**Table 1:** Frequency (‰) of Micronucleated cells (MN), Nucleoplasmic Bridges (NPB), apoptotic cells, and necrotic cells in zebra mussel haemocytes after exposure to different nominal doses of Zn(II): 1 mg/L/, 10 mg/L, 50 mg/L, 100 mg/L. (*) indicates significant differences respect to the control group.

	C	1 mg/L	10 mg/L	50 mg/L	100 mg/L
MN	0.95 ± 1.50	1.65 ± 2.06	1.10 ± 1.17	1.50 ± 1.29	1.95 ± 1.93 *
NPB	1.05 ± 1.96	2.50 ± 2.80	2.90 ± 4.09	3.00 ± 3.01	3.70 ± 4.57 *
Apoptosis	7.60 ± 10.88	8.90 ± 9.53	5.10 ± 3.70	13.44 ± 22.49	15.10 ± 14.29
Necrosis	0.00 ± 0.00	0.05 ± 0.22	0.00 ± 0.00	0.00 ± 0.00	0.00 ± 0.00

**Table 2:** Zn(II) concentration (mg/L) in zebra mussels’ exposure waters at time zero (T_0_) (soon after preparation and expoScheme 48 h (T_48_) for AFW (control); Zn(II) 50 mg/L in AFW; Zn-t CNS (Zn(II) 50 mg/L contaminated AFW treated with CNS), CNS (AFW treated with CNS only). Data are reported as mean ± SD from three independent experiments. (*) *p* < 0.05 respect to control within the same exposure time (#) *p* < 0.05 comparison between the two exposure times.

Exposure Group	Zn(II) T_0_ [mg/L]	Zn(II) T_48_ [mg/L]
AFW	0.022 ± 0.003	0.02 ± 0.014
Zn(II)	28.15 ± 6.84 *#	5.17 ± 3.60 *
CNS	0.183 ± 0.11	0.145 ± 0.12
Zn-t CNS	1.92 ± 0.39 #	0.45 ± 0.035

**Table 3:** Zn concentration (µg/g dry weight) in zebra mussels’ whole soft tissue after 48 h (T_48_) of exposure as follows: AFW (control); Zn(II) 50 mg/L in AFW; Zn-t CNS (Zn(II) 50 mg/L contaminated AFW treated with CNS, CNS (AFW treated with CNS only). Data are reported as mean ± SD. * *p* < 0.05; ** *p* < 0.01 in respect to the controls (ASW).

Exposure Group	Zn(II) T_48_ [µg/g]
AFW	121.36 ± 0.54
Zn (II)	505.50 ± 3.28 **
CNS	126.05 ± 0.44
Zn-t CNS	171.66 ± 0.44 *

**Table 4:** EC20 values after 5, 15, and 30 min of incubation and max effects recorded with the Microtox^®^ system. n.d. not detectable data being Max EC20(T48) after 30 min lower than 90%.

	T_0_				T_48_			
	EC20 (%)			Max Effect (%)	EC20 (%)			Max Effect (%)
	5 min	15 min	30 min	M ± SD	5 min	15 min	30 min	M ± SD
AFW	>90	>90	>90	−10.30 ± 2.91	>90	>90	>90	−10.46 ± 12.32
CNS	>90	>90	>90	0.75 ± 0.40	>90	>90	>80	n.d.
Zn-t CNS	>90	>90	>90	0.82 ± 10.12	>90	>90	>90	−16.91 ± 3.27

## Data Availability

Data are contained within the article.
